# Enzymatic digestion of articular cartilage results in viscoelasticity changes that are consistent with polymer dynamics mechanisms

**DOI:** 10.1186/1475-925X-8-32

**Published:** 2009-11-04

**Authors:** Ronald K June, David P Fyhrie

**Affiliations:** 1 Department of Cellular and Molecular Medicine, University of California, San Diego, La Jolla, USA; 2 Biomedical Engineering Graduate Group and Department of Orthopaedic Surgery, University of California, Davis, Sacramento, USA

## Abstract

**Background:**

Cartilage degeneration via osteoarthritis affects millions of elderly people worldwide, yet the specific contributions of matrix biopolymers toward cartilage viscoelastic properties remain unknown despite 30 years of research. Polymer dynamics theory may enable such an understanding, and predicts that cartilage stress-relaxation will proceed faster when the average polymer length is shortened.

**Methods:**

This study tested whether the predictions of polymer dynamics were consistent with changes in cartilage mechanics caused by enzymatic digestion of specific cartilage extracellular matrix molecules. Bovine calf cartilage explants were cultured overnight before being immersed in type IV collagenase, bacterial hyaluronidase, or control solutions. Stress-relaxation and cyclical loading tests were performed after 0, 1, and 2 days of incubation.

**Results:**

Stress-relaxation proceeded faster following enzymatic digestion by collagenase and bacterial hyaluronidase after 1 day of incubation (both *p *≤ 0.01). The storage and loss moduli at frequencies of 1 Hz and above were smaller after 1 day of digestion by collagenase and bacterial hyaluronidase (all *p *≤ 0.02).

**Conclusion:**

These results demonstrate that enzymatic digestion alters cartilage viscoelastic properties in a manner consistent with polymer dynamics mechanisms. Future studies may expand the use of polymer dynamics as a microstructural model for understanding the contributions of specific matrix molecules toward tissue-level viscoelastic properties.

## Background

Osteoarthritis is the most prevalent debilitating joint disease involving deterioration of the articular cartilage [1], yet the contributions of specific cartilage extracellular matrix molecules toward tissue-level viscoelastic mechanical properties are not sufficiently understood to enable specific osteoarthritis therapeutic strategies. Primary cartilage functions are mechanical: it provides low-friction surfaces for articulation [2-4] and deforms during joint contact to decrease contact pressure and increase joint stability [5]. Since cartilage deforms during *in vivo *loading, understanding the specific molecular origins of cartilage resistance to deformation is necessary to understand cartilage mechanical function. This study examines the molecular origins of cartilage viscoelastic deformations in the context of polymer dynamics.

The cartilage extracellular matrix is primarily composed of two polymers [6]: collagen and the proteoglycan aggregate. The major collagen in cartilage is type II, and it forms a fibrillar copolymer also containing collagen types IX and XI [7]. The proteoglycan aggregate consists of the glycosaminoglycans keratin and chondroitin sulfate which are covalently bound to the protein aggrecan which non-covalently binds with hyaluronan [8].

Previous research has separated cartilage viscoelasticity into flow-dependent and flow-independent regimes [9-11]. Many studies have examined the role of fluid flow [12-16]. However, few studies have examined flow-independent viscoelasticity in cartilage. This study examined polymer dynamics as a mechanism of cartilage flow-independent viscoelasticity.

For cartilage, polymer dynamics is defined as the motions and interactions of entangled polymers such as those of the cartilage extracellular matrix. As such, polymer dynamics may be a mechanism of cartilage flow-independent viscoelasticity [9,10]. Because polymer dynamics is based on molecular-level interactions, investigation of cartilage mechanics using polymer dynamics may elucidate specific molecular contributions toward tissue-level mechanical properties that have not been previously recognized. Such an understanding may yield novel therapies and tissue engineering strategies for osteoarthritis

The theory of polymer dynamics quantitatively describes the motions and interactions of polymer molecules [17]. A fundamental concept in polymer dynamics is that the mechanical properties of entangled polymers depend on the polymer molecular length [18]. Polymer dynamics theory predicts that the stress-relaxation time constant decreases with decreased average polymer molecular weight, with the quantitative relationship defined by the specific type of polymer motion [19]. For cyclical loading, polymer dynamics predicts that both the storage and loss modulus will decrease with decreased polymer molecular length. We hypothesized that (a) cartilage stress-relaxation will proceed faster and (b) cartilage storage and loss moduli will decrease after incubation with enzymes which are expected to shorten cartilage extracellular matrix polymers.

The use of selective enzymatic digestion is one method for examining the specific biomechanical contributions of cartilage extracellular matrix molecules: changes in mechanical properties following enzymatic cleavage imply functional relevance of the enzyme substrate. Previous studies have revealed that enzymatic digestion changes various cartilage mechanical properties [20-25]. In this study, after overnight tissue culture, samples of bovine cartilage were mechanically tested followed by incubation in control solutions or solutions containing collagenase or bacterial hyaluronidase with repeated mechanical testing after 1 and 2 days of incubation.

Stress-relaxation proceeded faster following enzymatic digestion by collagenase and bacterial hyaluronidase after 1 day of incubation as indicated by smaller time constants (both *p *≤ 0.01). The storage and loss moduli at frequencies of 1 Hz and above were smaller after 1 day of digestion by collagenase and bacterial hyaluronidase (all *p *= 0.02). These results demonstrate that enzymatic digestion alters cartilage viscoelastic properties in a manner consistent with polymer dynamics mechanisms and further implicate polymer dynamics as a flow-independent mechanism of cartilage viscoelasticity [26-28].

## Methods

### Sample Harvest and Tissue Culture

**Figure 1:**
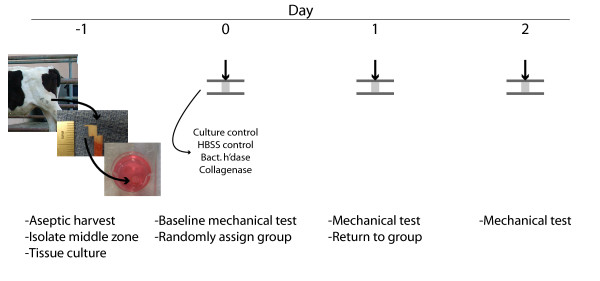
**Experimental Protocol**. Middle-zone patellofemoral bovine calf cartilage explants were harvested and cultured overnight. Samples were subjected to baseline mechanical testing and randomly assigned to an enzyme- treatment or control group. Mechanical testing was performed again after 1 and 2 days of exposure to the treatment.

Bovine cartilage explants (diameter: 3.32 ± 0.01 mm) were aseptically harvested from 1-3 month calf stifle joints and maintained in tissue culture or enzyme solutions during the experimental timecourse (Figure 1). Samples were harvested from a standard location on the lateral patellofemoral groove, midway between the proximal and distal boundaries and placed in Dulbecco's Modified Eagle's Medium (DMEM) containing penicillin and streptomycin. The surface and deep zones of the osteochondral explants were then removed to obtain middle-zone explants of a standard height (3.99 ± 0.04 mm) [29]. Middle-zone explants were rinsed 3 times with DMEM and antibiotics, and incubated overnight in a chemically-defined culture medium at 37°C in 5% CO_2_. The chemically-defined medium contained 0.1% bovine serum albumin (BSA), insulin-transferrin-selenium (ITS) (1 mg/mL, 0.55 mg/mL, and 0.67 mg/mL, respectively), 50 μg/mL L-ascorbic-acid-2-phosphate, 100 U/mL penicillin, and 100 μg/mL streptomycin.

### Enzyme Treatment

To assess the role of specific enzymatic degradation in cartilage mechanics, samples were subjected to enzymatic digestion by 5 U/mL Type IV Collagenase (n = 12 samples) (Sigma-Aldrich, St. Louis, Missouri, USA., C5138) or 50 U/mL Bacterial Hyaluronidase (n = 9) (Sigma-Aldrich, St. Louis, Missouri, USA., H1136). The enzyme concentrations were based on preliminary experiments that determined concentrations which resulted in measurable changes in biomechanical properties during the experiment. The enzyme solvent was Hank's Balanced Saline Solution (HBSS) with 0.01% BSA as a protein carrier. After overnight equilibration in tissue culture, samples were immersed in HBSS for 1 hour prior to initial mechanical testing at 37°C. After mechanical testing, samples were placed in the appropriate enzyme solution and incubated at 37°C. Mechanical testing was performed after 1 and 2 days of incubation in the enzyme solutions (Figure 1).

Two control groups were used to assess changes in mechanical properties throughout the experiment. The first group, HBSS-control (n = 11), was incubated in the HBSS solution (HBSS with 0.01%BSA) in the absence of enzymes. The second group, Culture-Control (n = 12), was incubated in tissue culture medium and immersed in HBSS for 1 hour prior to mechanical testing. These samples were subjected to the same timecourse of mechanical testing as described above.

### Viscoelastic Testing

Mechanical testing consisted of unconfined compression stress-relaxation followed by cyclical loading in an Enduratec ELF 3200 uniaxial testing system. Prior to testing, the sample diameter was measured using digital calipers. Samples were placed on a polished stainless steel loading platen in a heated loading chamber. A polished stainless steel platen was lowered at 0.05 mm/sec until a 60 kPa prestress was achieved. This prestress was allowed to relax for 4 minutes during which time the bath was filled with warmed HBSS and temperature control was initiated. The initial specimen height was defined as the height at which the preload was achieved.

Stress-relaxation tests were performed by applying a 5% nominal compressive strain using a rapid platen displacement rate of 10 mm/sec. After 600 seconds, small-amplitude cyclical strains (~0.5%) were applied at frequencies of 0.01, 0.1, 1, 10, and 20 Hz for at least 4 cycles. During stress-relaxation, force data were collected at 180 Hz, and, for cyclical loading, sampling occurred at a minimum of 20 times the loading frequency. Apparent stresses were calculated by dividing the compressive force by the initial sample cross-sectional area. 600 seconds of stress relaxation provided a compressive equilibrium such that linear regression of the final 100 points of stress-time data found no significant slope in any dataset at the 99% confidence interval.

### Data Analysis

The stress-relaxation data were fit with a stretched-exponential model that has been linked [30] to polydisperse polymer systems such as cartilage:(1)

*σ*_*peak *_and *σ*_*eq *_are the peak and equilibrium stress, respectively, which are defined by the experimental data. *τ *is the time constant of stress-relaxation, which is related to the physical characteristics of the polymer system (*e.g*. temperature, polymer length, and concentration) [18]. *β *is the stretching parameter, related to the specific type of polymer motion (*e.g*. reptation). *τ *and *β *were determined using nonlinear curvefitting. In addition to *τ *and *β*, a model-independent parameter, , was used to quantify stress-relaxation (Figure S1 [see Additional file 1]) [26-28].

Cyclical loading experiments were analyzed by finding the phase lag of the stress-relative to the strain. Storage and loss moduli were calculated as the in-phase and out-of-phase amplitude ratios of stress to strain, respectively. For frequencies greater than 0.1 Hz, we observed nonlinear cyclical behavior demonstrated by time-variant stress-responses during the first few loading cycles. In these cases, the storage and loss moduli were determined from the steady-state portion of the curves.

### Statistics

To compare the effects of treatment time and group, statistical analysis was performed using repeated-measures ANOVA with an *a priori *significance level of 0.05. The independent variables for the statistical analysis were the time (Days 0, 1, and 2) and treatment (HBSS-control, Hyaluronidase, Collagenase, Culture-control). The dependent variables were the results of the mechanical testing *σ*_*peak*_, *σ*_*eq*_, *τ*, *β*, and  for stress relaxation and storage and loss modulus for cyclical loading. Planned comparisons were made between the enzyme-treatment groups and the HBSS-control group after 1 and 2 days of culture. To determine if further digestion resulted in additional mechanical property changes, Bonferroni *post hoc *comparisons [31] were made between days 0 and 1 and days 1 and 2 for enzyme-treated groups for which planned comparisons were significant. Results are expressed as mean ± standard error.

## Results

**Figure 2:**
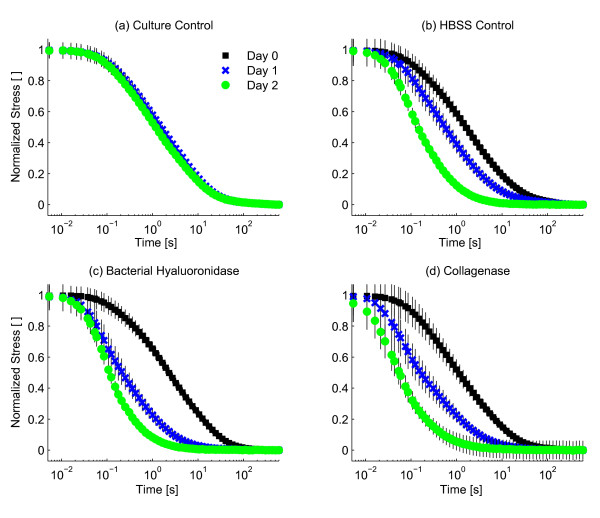
**Stress-relaxation proceeds faster with enzymatic digestion**. Stress-relaxation data were normalized to a range between 1, representing the peak stress and 0 representing the equilibrium stress. Stress-relaxation proceeded faster after both enzymatic digestion (c-d) and incubation in HBSS (b). No major changes in the rate of stress-relaxation were observed in control samples samples maintained in tissue culture (a). Marked changes in the rate of stress-relaxation were observed after 1 day of incubation in bacterial hyaluronidase. Bars represent ± sem.

**Figure 3:**
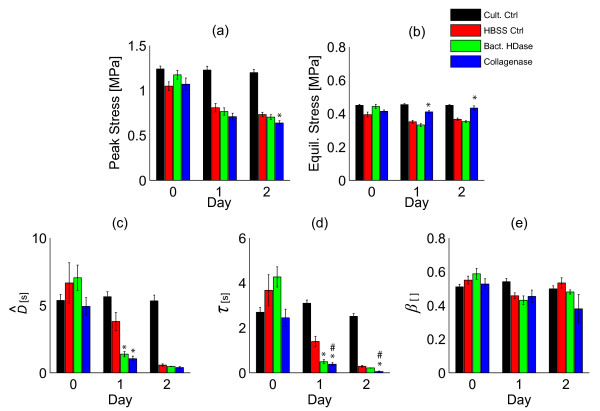
**Enzymatic digestion results in changes in cartilage stress-relaxation**. Significant decreases in  and *τ *compared with HBSS controls indicate that stress-relaxation proceeded faster in enzymatically-digested samples than in HBSS controls after 1 day of treatment. , peak stress, and equilibrium stress were calculated directly from the experimental data, and *τ *and *β *were determined by nonlinear curvefitting of the stretched exponential model to the stress-relaxation data. *indicates *p *≤ 0.01 compared to HBSS-control at the same day. # indicates *p *< 0.01 within a group between days 1 and 2 using Bonferroni Post-hoc tests.

Stress-relaxation proceeded faster in samples subjected to enzymatic digestion (Figures 2, 3 S3 [see Additional file 1], Table 1). The model-independent parameter  and the stretched exponential time constant *τ *were smaller for bacterial hyaluronidase and collagenase than for the HBSS control samples after 1 day of enzyme exposure (*p *< 0.01, Figures 3c, 3d, and S3 [see Additional file 1]). *Post hoc *tests showed that additional digestion further reduced *τ *(*p *< 0.01, Figure 3d). For collagenase samples, the equilibrium stress was larger than for controls after 1 and 2 days of digestion (*p *< 0.01, Figure 3b). The stretched exponential model described the stress-relaxation data well (*R*^2 ^= 0.900 ± 0.012, Figure S2 [see Additional file 1]). The initial heights of samples in the culture control group at days 0, 1, and 2 were 4.05 ± 0.05, 4.09 ± 0.04, and 4.11 ± 0.04, respectively, suggesting that mechanical testing did not alter normal tissue growth and swelling [32].

**Table 1:** Stress-Relaxation Results, Day 1

	*σ*_*peak*_	*σ*_*eq*_		*τ*	*β*	R^2^
	[kPa]	[kPa]	[sec]	[sec]	[]	[]
HBSS Control	808 ± 43	351 ± 8	3.80 ± 0.62	1.40 ± 0.21	0.46 ± 0.02	0.936 ± 0.011
Bacterial Hyaluronidase	766 ± 36	332 ± 7	1.39 ± 0.16*	0.49 ± 0.08	0.43 ± 0.02	0.949 ± 0.006
Collagenase	709 ± 34	410 ± 8*	1.05 ± 0.17*	0.38 ± 0.07	0.45 ± 0.03	0.864 ± 0.022
Culture Control	1227 ± 39	453 ± 5	5.65 ± 0.34	3.10 ± 0.13	0.54 ± 0.02	0.972 ± 0.002

Mean ± SEM. * indicates significant difference between enzyme group and HBSS control.

**Figure 4:**
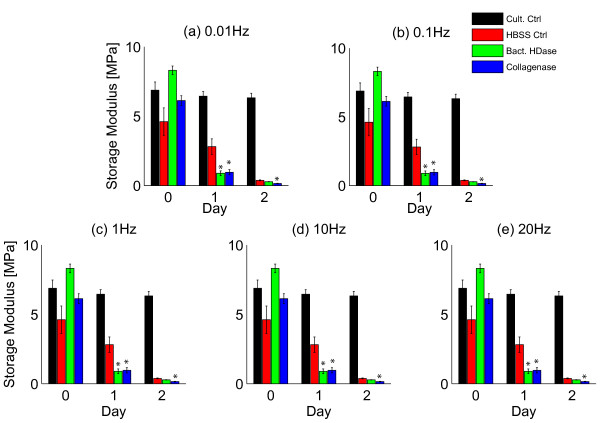
**Decreased storage moduli following enzymatic digestion**. The storage modulus decreased at all tested frequencies following hyaluronidase and collagenase digestion. After 1 day of treatment, bacterial hyaluronidase and collagenase had significant effects compared to HBSS controls at all frequencies (all *p *≤ 0.02). *indicates *p *≤ 0.02 compared to HBSS-control at the same day.

**Figure 5:**
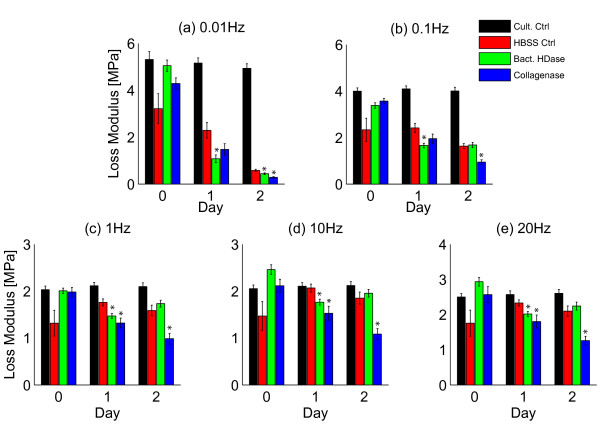
**Decreased loss moduli following enzymatic digestion**. Decreases in loss modulus were observed at most tested frequencies following hyaluronidase and collagenase digestion. After 1 day of treatment, the bacterial hyaluronidase group had smaller loss moduli at all frequencies than the HBSS-control group (all *p *< 0.01). After 1 day of treatment, the collagenase group had smaller loss moduli at frequencies greater than 0.1 Hz (all *p *< 0.02). *indicates *p *≤ 0.02 compared to HBSS-control at the same day.

Cyclical loading showed that enzymatic digestion decreased both storage and loss moduli relative to HBSS-controls at most frequencies (Figures 4 and 5, Table 2). For both bacterial hyaluronidase and collagenase, the storage modulus was significantly smaller than controls after 1 day of digestion at all frequencies (*p *≤ 0.01, Figures 4 and 5). For bacterial hyaluronidase the loss modulus decreased relative to controls at all frequencies after 1 day (*p *≤ 0.01, Figure 5). For collagenase, the loss modulus was significantly smaller relative to controls at frequencies greater than 0.1 Hz (*p *≤ 0.01, Figure 5c-e).

**Table 2:** Cyclical Loading Results, Day 1

	Loading Frequency [Hz]									
	0.01		0.1		1		10		20	
[MPa]	E'	E"	E'	E"	E'	E"	E'	E"	E'	E"
HBSS Control	2.81 ± 0.54	2.29 ± 0.32	6.09 ± 0.89	2.41 ± 0.19	8.06 ± 0.99	1.73 ± 0.07	10.11 ± 1.08	2.05 ± 0.08	11.15 ± 1.09	2.30 ± 0.08
Bacterial Hyaluronidase	0.89 ± 0.15*	1.08 ± 0.14*	2.77 ± 0.30*	1.66 ± 0.88*	4.32 ± 0.33*	1.45 ± 0.05*	5.93 ± 0.39*	1.74 ± 0.05*	6.81 ± 0.38*	2.02 ± 0.05*
Collagenase	0.96 ± 0.17*	1.47 ± 0.22	3.43 ± 0.47*	1.95 ± 0.18	4.77 ± 0.56*	1.30 ± 0.09*	5.81 ± 0.63*	1.52 ± 0.14*	6.42 ± 0.69*	1.81 ± 0.17*
Culture Control	6.44 ± 0.30	5.16 ± 0.20	14.09 ± 0.47	4.09 ± 0.12	17.29 ± 0.56	2.08 ± 0.07	19.5 ± 0.65	2.1 ± 0.07	20.23 ± 0.70	2.58 ± 0.10

E' represents storage modulus and E" represents the loss modulus. * indicates significant differences between enzyme-treated group and HBSS control group.

Loading frequency had significant effects on both storage and loss moduli (Figure 4, 5). The storage modulus increased with each frequency increment in all groups after at all timepoints (all *p *< 0.01) except for day 0 in the collagenase group between frequencies of 1 and 10 Hz and 10 and 20 Hz (*p *= 0.28 and 0.07, respectively). The loss modulus at 20 Hz was higher than at 10 Hz for all treatment groups on all days (all *p *< 0.01). The loss modulus was also larger at 10 Hz than at 1 Hz for all groups on all days (all *p *< 0.01) except for the culture control group on days 1 and 2 (*p *= 0.26 and 0.03, respectively) and for the collagenase group on day 0 (*p *= 0.64).

...

## Discussion

These experiments tested the prediction from polymer dynamics theory that changes in cartilage biopolymer length would result in changes in cartilage viscoelastic properties. Polymer dynamics theory predicts decreases in stress-relaxation time constant, storage and loss modulus as a result of decrease in average molecular length [18]. Bacterial hyaluronidase and Type IV collagenase are known to decrease average molecular length by cleaving molecules of the cartilage extracellular matrix. Type IV collagenase cleaves the collagen triple-helix at multiple locations [33]. The bacterial hyaluronidase used in these experiments has been demonstrated to specifically cleave hyaluronan and not the other cartilage glycosaminoglycans of chondroitin and keratin sulfate [34]. Although substantial changes in viscoelastic properties resulted from enzymatic digestion, one limitation of this study is that quantification of the size distributions of the relevant macromolecules was not possible because of the small size of the samples.

Enzymatic digestion had marked effects on cartilage viscoelasticity which are consistent with polymer dynamics theory. After 1 day of enzyme treatment, we observed marked decreases in the stress-relaxation parameters of  and *τ*, as well as decreases in storage moduli at all frequencies and loss moduli at frequencies greater than 0.1 Hz. These changes are consistent with the predictions of polymer dynamics theory and support polymer dynamics as a flow-independent mechanism of cartilage-viscoelasticity. The complete theory of polymer dynamics has been derived using statistical physics [18]. Mathematical modelling of cartilage mechanics via polymer dynamics is theoretically possible once the specific types of polymer motions and distributions of molecular lengths are known. Future research into polymer dynamics in cartilage may determine what specific types of motion (*e.g*. Rouse, reptation, etc.) cartilage polymers undergo. Therapeutic opportunities based on polymer dynamics may be possible. For example, if the dynamics of a specific cartilage polymer (*e.g*. hyaluronan) were found to be paramount to cartilage mechanical function, tissue engineers could specifically tailor their scaffolds to include these dynamics.

Interpretation of the effects of specific enzymes should be done with caution as cartilage composition is heterogeneous. Collagenase digestion may affect the behavior of the proteoglycan aggregate which is thought to be entangled within the collagen network [35]. A disrupted collagen network may increase mobility of the proteoglycan aggregate which polymer dynamics theory predicts to decrease the stress-relaxation time. This interpretation is supported by the observed equilibrium stress increase upon collagenase digestion (Figure 3b, Table 1). Conversely, digestion of the proteoglycan aggregate (*e.g*. hyaluronan by bacterial hyaluronidase) might not affect the behavior of the collagen network due to the fibrillar, crosslinked nature of cartilage collagen [36]. The absence of changes in both peak and equilibrium stress due to hyaluronidase digestion supports this interpretation.

Collagenase digestion accelerated stress-relaxation and increased the equilibrium stress (Figures 2, 3, Table 1). Collagenase digestion also decreased the storage modulus at all frequencies and the loss modulus at frequencies of 1 Hz and higher. These effects may be due to (a) altered collagen behavior or (b) altered behavior of another cartilage component induced by collagenase digestion. The observed increase in equilibrium stress upon collagenase digestion is not consistent with collagenase digestion affecting only collagen behavior, assuming that a digested collagen network is weaker than an intact one. Previous research suggests that proteoglycan aggregate swelling is restricted by collagen, and the observed collagenase-induced increase in equilibrium stress may be a result of reduced proteoglycan aggregate constraint [37]. Reduced proteoglycan aggregate constraint will also lead to accelerated stress-relaxation. These experiments cannot separate contributions due to altered collagen behavior and decreased proteoglycan constraint on the collagenase-induced decreases in  and *τ*. However, both interpretations are consistent with polymer dynamics as a mechanism of cartilage viscoelasticity.

Bacterial hyaluronidase treatment resulted in accelerated stress-relaxation as shown by decreased stress-relaxation time constant and  (Figure 3, Table 1) and decreased storage and loss moduli at all frequencies after 1 day of digestion (Figures 4, 5, Table 2). Bacterial hyaluronidase is known to cleave only hyaluronan [34] so we attribute these effects to decreases in average hyaluronan length caused by enzymatic cleavage. These results are consistent with the polymer dynamics prediction: shorter molecules result in faster stress-relaxation and decreased storage and loss moduli.

These results are also consistent with a previous microstructural model of equilibrium cartilage mechanics in which collagen fibers are considered to constrain the swelling of the proteoglycan aggregate [37]. The observation that collagenase digestion increased the equilibrium stress further supports this concept, suggesting that weakened collagen allows further swelling of the proteoglycan aggregate which increases the equilibrium stress.

One limitation of this study is that the diffusion of enzyme from the solution to the cartilage tissue is inherently limited due to restricted diffusivity within the cartilage [38,39]. This will result in spatially heterogeneous enzymatic digestion within the cartilage explants. Consequently, the mechanical testing results will reflect changes in average molecular length since molecules toward the center of the sample are less likely to be digested than those at the perimeter. This biases the results toward the HBSS-controls, suggesting that spatially homogeneous digestion with these enzyme concentrations would likely result in greater changes than those presently observed.

Maintaining cartilage in HBSS will alter chondrocyte biology, as the solution contains no glucose or ATP which are needed to fuel cellular processes [40]. We used two sets of control samples (HBSS and culture controls), and the results show degradation of the HBSS-control mechanical properties relative to those of the culture-controls with increased incubation time. This background degradation due to HBSS may result from the release of proteolytic enzymes. If some of these enzymes are collagenases or hyaluronidases, the actual enzyme concentration to which the samples were exposed might have been slightly higher than that obtained by diffusion alone. Enzymatic digestion resulted in significantly more degradation than that observed in the HBSS-control samples, indicating that the experimentally-induced enzymatic effects were greater than the biological effects induced by incubation in HBSS alone. Furthermore, the half-lives of collagen and aggrecan are estimated to be on the order of years [41-43]. Therefore, relatively small changes in matrix content are expected unless dramatic catabolism were induced by HBSS.

The unconfined compression test has substantial advantages over other testing configurations because it is unbiased, so both flow-dependent and flow-independent processes can occur. Conversely, a confined compression test is biased toward the effects of flow, and pure shear tests are biased toward the effects of flow-independent processes.

To analyze the potential role of fluid flow in these experiments we compared the stress-relaxation time constant to the estimated fluid-flow time constant and examined the frequency-dependent phase lag of our cyclical loading data. The time constant for unconfined compression stress-relaxation due to fluid flow [12] is defined by the sample radius, *r*, the tensile equilibrium aggregate modulus, *H*_+*a*_, and the hydraulic permeability, *k *[44]:(2)

**Figure 6:**
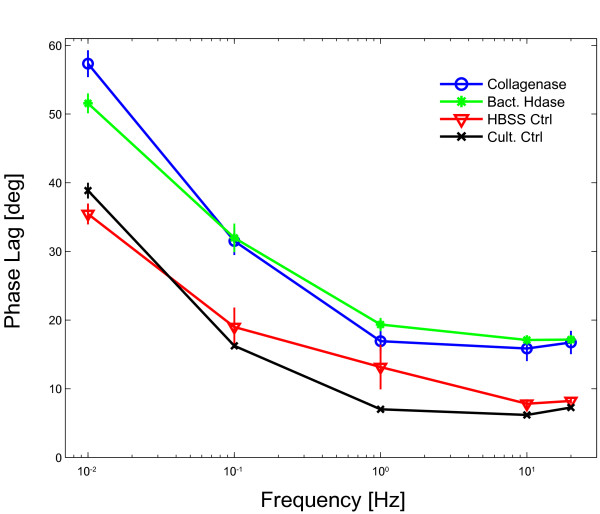
**Phase lag data suggest flow-independent processes**. The phase lag data suggest that cyclical loading experiments probed flow-independent processes after 1 day of digestion. Previous research [9,10] suggests that flow-dependent viscoelasticity is frequency-dependent with substantial dissipation, as measured by the cyclical loading phase lag between the applied displacement and the measured stress, between 10^-6 ^and 10^-1 ^Hz. The present cyclical loading data found substantial dissipation at frequencies of 10^-2 ^Hz and higher, indicating that these data represent flow-independent processes.

Previous modeling results find that collagenase digestion results in *k*~3.8 × 10^-15 ^m^4^(N-s)^-1 ^and *H*_+*a*_~2.5 MPa [45]. The present values of *τ *and  are much smaller than the estimated fluid-flow time constants (Table 3), suggesting that the observed results and model parameters are representative of flow-independent processes. Previous studies find that the phase lag for cartilage fluid flow in unconfined compression becomes negligible at frequencies above 0.01 Hz [9,10]. However, our results (Figure 6) have substantial phase lags at all tested frequencies, further supporting our flow-independent interpretation of the data.

**Table 3:** Fluid flow dynamics are much slower than those observed in the present experiments.

[sec]	*τ*		*τ*_*Flow*_
Collagenase	0.38 ± 0.07	1.05 ± 0.17	237 ± 3

The fluid flow time constants were estimated from model parameters resulting from previous biomechanical studies of enzymatically digested cartilage [14,35].

## Conclusion

The theory of polymer dynamics predicts that the stress-relaxation time constant, the storage modulus, and the loss modulus will all decrease with decreases in average molecular length. In this study, exposure of cartilage explants to solutions containing Type IV collagenase and bacterial hyaluronidase resulted in decreases in stress-relaxation time constant, storage modulus, and loss modulus, relative to control samples maintained in enzyme-free solutions. The results support polymer dynamics as a conceptual model for understanding cartilage flow-independent viscoelasticity.

## Competing interests

The authors declare that they have no competing interests.

## Authors' contributions

RKJ performed the experimental work, numerical modeling, statistical analysis, and participated in writing the manuscript. DPF advised with study design and participated in writing the manuscript.

## Supplementary Material

Additional file 1 **Supplementary Figures**. This file contains the supplementary figures for the manuscript. Click here for file
