# Supplementary material for: Enzymatic digestion of articular cartilage results in viscoelasticity changes that are consistent with polymer dynamics mechanisms
Source: Biomed Eng Online. 2009 Nov 4;8:32. doi: 10.1186/1475-925X-8-32 (PMC2778644; doi:10.1186/1475-925X-8-32)
Supplement: Additional file 1 — Supplementary Figures. This file contains the supplementary figures for the manuscript. [file 1475-925X-8-32-S1.pdf]

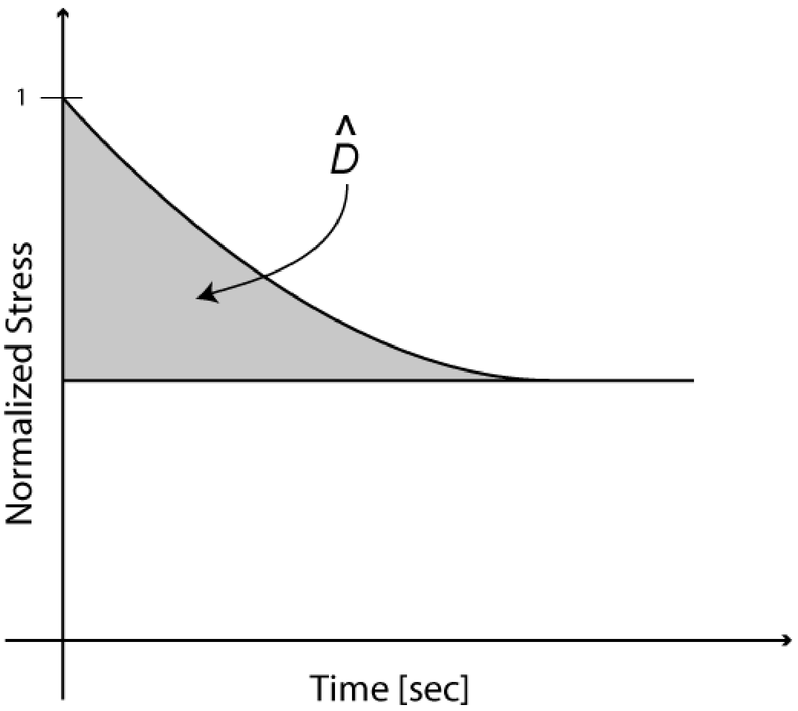

Fig. S1

Model-Independent Measure of Stress-relaxation. The parameter $\hat{D}$ was used as a model-independent measure of stress-relaxation, calculated by determining the area under the normalized stress-relaxation curve shown in gray in the diagram. $\hat{D}$ has units of time and is analogous to a time constant: larger values indicate slower stress-relaxation and smaller values indicate faster stress-relaxation.

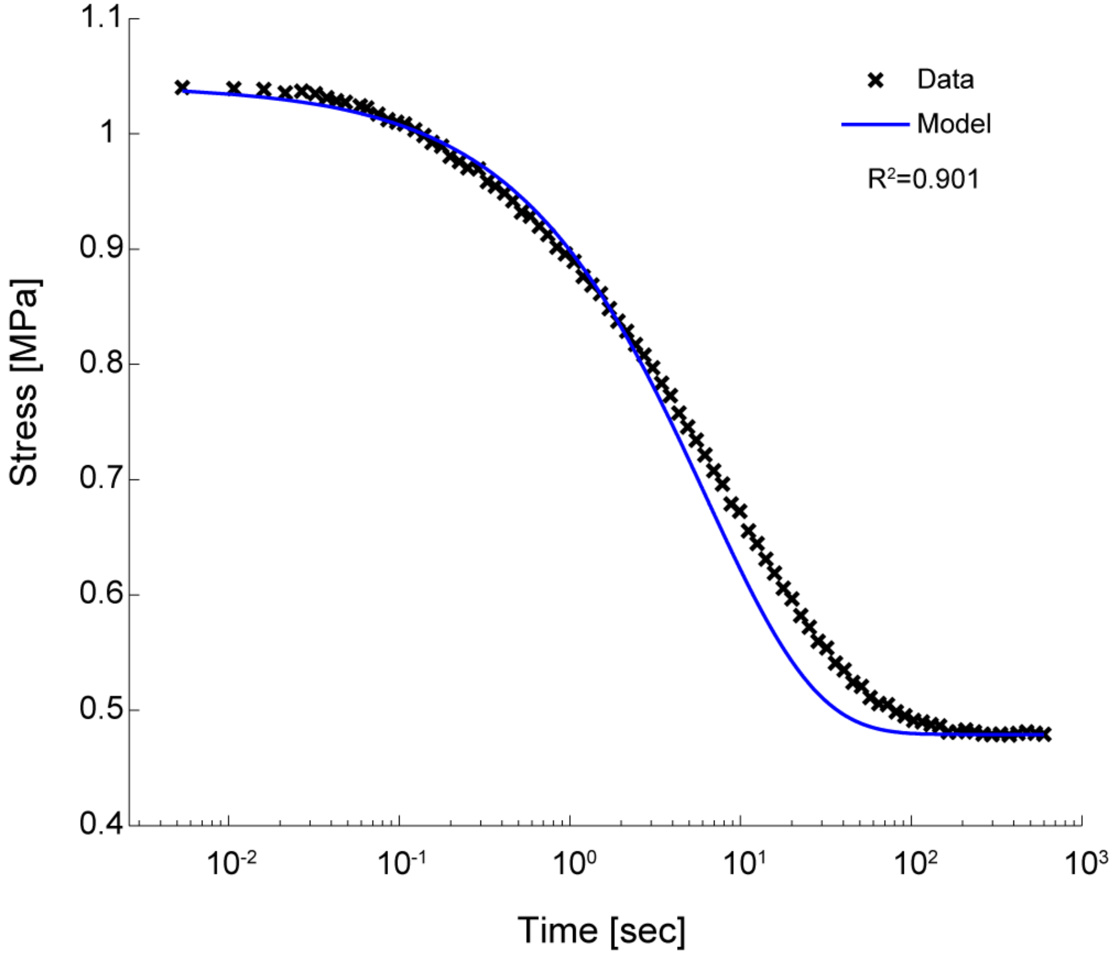

Fig. S2
Representative Stretched Exponential Model fit, R^2=0.901. Overall, the stretched exponential model described the data well, R^2=0.900±0.012.

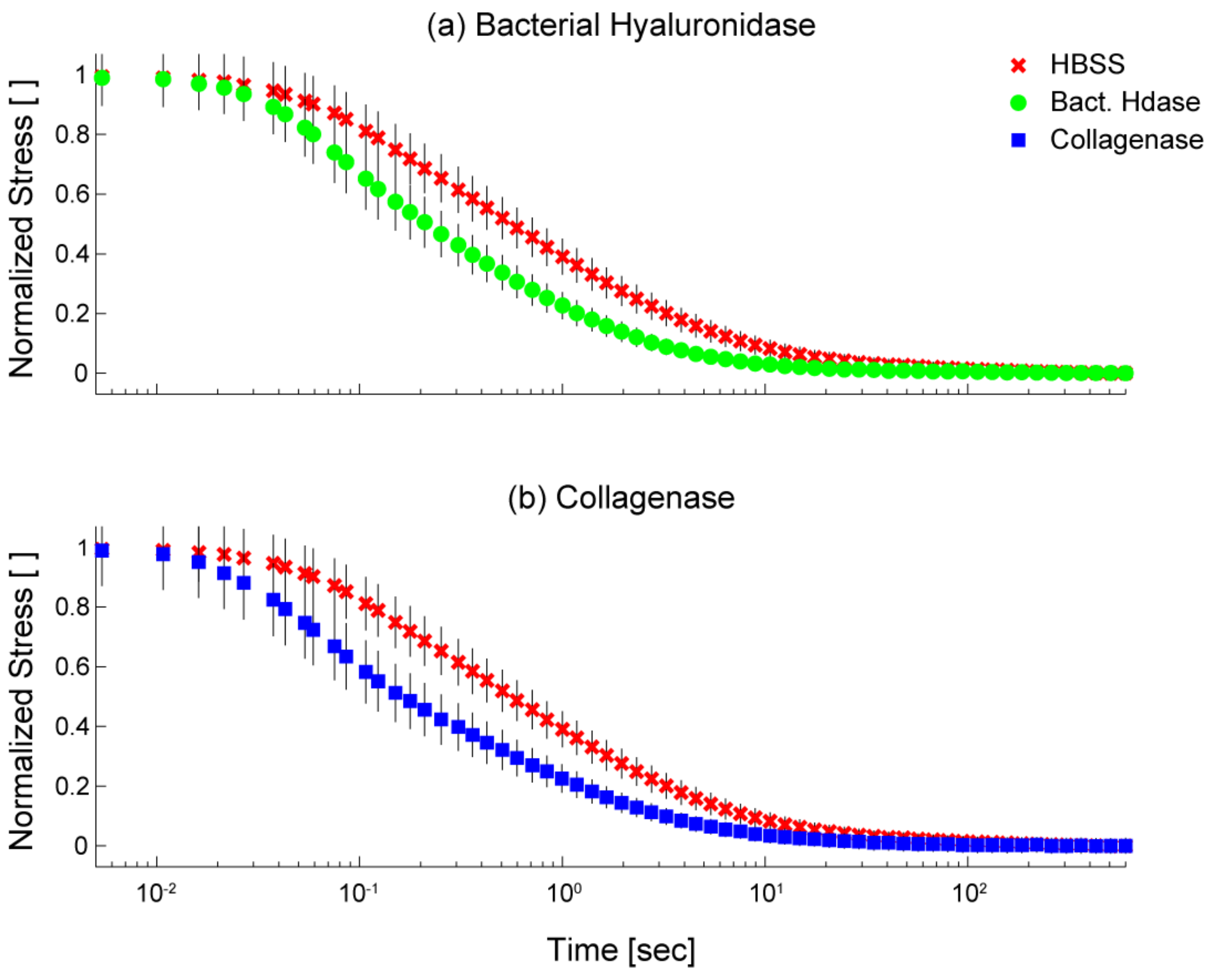

Fig. S3

Normalized Stress-Relaxation Data, Day 1, Compared with HBSS Controls. Both collagenase and bacterial hyaluronidase resulted in faster stress-relaxation than HBSS alone.
